# The Social Construction of the Child’s Body and Its Relationship with Football Practice

**DOI:** 10.3390/sports13070202

**Published:** 2025-06-23

**Authors:** Gil-Madrona Pedro, Losada-Puente Luisa, Ruiz-Izquierdo Javier, Martínez-López María, Morcillo-Martínez Antonio

**Affiliations:** 1 Department of Didactics of Physical, Artistic and Music Education, Faculty of Education of Albacete, University of Castilla-La Mancha, 02071 Albacete, Spain; pedro.gil@uclm.es (G.-M.P.); antonio.morcillo@uclm.es (M.-M.A.); 2 Specific Didactics and Methods of Research and Diagnosis in Education Department, Faculty of Education Sciences, Universidade da Coruña, 15008 Coruña, Spain; luisa.losada@udc.es; 3 Faculty of Education of Albacete, University of Castilla-La Mancha, 02071 Albacete, Spain; javier.ruiz18@alu.uclm.es; 4 Department of Pedagogy, Faculty of Education of Cuenca, University of Castilla-La Mancha, 010107 Cuenca, Spain; 5 Department of Pedagogy, Faculty of Education of Albacete, University of Castilla-La Mancha, 02071 Albacete, Spain

**Keywords:** body image, child body, soccer, physical activity, body perception, physical appearance

## Abstract

Body image is understood as a social construction acquired over the years that is generated as people learn behaviors and relate to each other in the search of their own identity. The aim of this research is to identify the personal and contextual factors which may influence the positive social, negative social, and self-concept dimensions of the social construction of body image on boys and girls from fifth and sixth grades of Primary Education by developing a descriptive exploratory study. For the sample, 719 pupils of fifth grade and sixth grade from Primary Education were selected to develop this research; 373 of them were boys (51.9%) and 340 girls (47.3%), with 6 lost samples. The main results of this research suggested associations between the lack of physical activity, the increase in BMI, and the increase in the number of siblings who worried more about body perception. Likewise, football stands out after detecting links between the practice of this sport and influences on body perception and self-perception.

## 1. Introduction

Body image is a complex [1] and multifaceted construct because it involves social, biological, and psychological aspects [2,3]. Even when there is an absence of scientific consensus when defining this concept, the contributions of authors [2] such as Raich et al., (1996) [4], Slade (1994) [5], or Thompson (1990) [6] refer to it as follows: ‘*self-perception of the body image generates a mental representation, composed of a perceptual body schema, as well as associated emotions, behaviours and thoughts*’ (p. 8).

The dynamism developed from this construct transcends not only its definition, but also its concrete factors, which affect the process of constructing body image. There are some factors of sociocultural risk such as media and social environment (composed by families and groups of friends) that have been highlighted as sociocultural risk factors that determine the self-perception of body perception at young ages [7,8]. The sociocultural pressure developed from imposing an aesthetic model can lead to an increased preoccupation with all things related to body and social comparison; a process significantly correlated with various mediations of negative body image. Likewise, over the years, women have been considered more critical in relation to judgments about physical appearance and attractiveness. However, as of today, beyond the normalization of female body dissatisfaction, it is noteworthy that this same feeling of body discrepancy is growing considerably in men [7,9]. 

Another factor with a huge influence on body image is physical activity. Its practice has been consolidated as a recognized field of vital interest for the scientific community due to its great potential to promote physical and psychological health [10]. Researchers such as Sabiston et al. (2019), after a bibliographic review that included more than 200 publications on this topic, considered body image as an antecedent and consequence of physical activity and sports practice [11]. Furthermore, researchers found a relationship between these factors and improvement on body image [11,12,13]. This idea is reinforced after finding a direct relationship between the lack of physical activity and body dissatisfaction [14,15] and an inadequate body perception [16].

Body image is highly connected to a wider construct, which also includes different perceptions of the body shape or the subject’s own sense of competence: the physical self-concept [1]. That is why understanding how it works, and which elements play an active role in developing a positive or a negative body image, is crucial at young ages. One of the most important factors is sports practice as it is a relevant factor which can influence teenagers’ body image both positively and negatively even at pre-teen stages [17]. However, finding balance for sports practice and well-being is key for children’s and teenagers’ body development [18]. This research conducted by Bhati et al. (2022) shows the importance of sports practice for a correct developmental evolution of features such as body image and posture, both regulated and improved while practicing physical activity [18].

Body image is such a determinant factor that might influence health and well-being over lifestyle, habits, and even hobbies. Having said that, body image is strongly related to Body Mass Index too, as it is related to health and can be stigmatized sometimes [19]. Empirical evidence underscores the importance of sports practice on body perception and cognitive development among children and adolescents. Zacagni et al. (2023) concluded that regular engagement in physical activity is associated with increased body satisfaction and the adoption of more realistic and healthy body ideals [19]. Complementing this evidence, Toselli et al. (2022) highlighted how gender and the type of sport practiced can influence more positive self-perceptions by finding variations between genders and modalities [20].

Furthermore, it has been demonstrated that sports practice involves cognitive demands, especially in the practices that require strategic decision making, which are positively correlated with the development of executive functions, including attention regulation, inhibitory control, and planning skills [21]. Collectively, this research provides a robust theoretical foundation for the integration of structured sports programs that can help to improve body image and its perception without forgetting how complex the conceptualization of body image is. This complex mental representation is the key for the psychological well-being and self-evaluation of the subject, which depend on four subdomains that have a determining influence on the configuration of the self-concept and the construction of body image: sports competence, physical condition, strength, and physical attractiveness [22].

The control and execution of basic motor skills (sport competence), which represent the alphabet of motor skills [23], are a crucial aspect in the development of body image at early ages. This is due, on the one hand, to the relevance acquired by the processes of social comparison and the influence of peer groups on the individual’s self-perception [24] and, on the other hand, to the relationship found between body dissatisfaction and low levels of physical competence [20] or worse perceptions about sports skills [1], as well as a notable deterioration of body image and sense of competence in relation to the control of locomotor skills such as running or jumping, in overweight children or preadolescents [25]. This being so, the lack of mastery of physical and motor skills represents a risk factor that can damage the body image of the young as it is linked to their posture and balance [18].

Positive body self-perception is also related to the level of physical fitness, while body dissatisfaction is a barrier to sport participation and offers a worse result in fitness level, while improvement in physical fitness leads to a more positive perception of body image, especially in women [1,26]. This level of body satisfaction can be determined, in turn, by the level and perception of strength [1,25] or by the body composition of the individual [14]. Several studies find strong associations between body dissatisfaction and Body Mass Index (BMI) in adolescents [27,28], as well as a more negative subjective perception of their weight and a lower valuation of their body image and physical self-concept [29].

Physical appearance is defined as an aspect of great relevance in the development of general self-concept [1], body image [28], and more, considering preadolescence as a stage where social approval of physical appearance plays a fundamental role in achieving body satisfaction, self-esteem, and mental health [19,26]. In this same line, ref. [30] defended that those modifications in body shape and appearance of character inherent to PA entail a positive influence on body image and, furthermore, Ref. [29] stated that “physical activity exerts a significant indirect effect on the subjective importance of physical appearance” (p. 31). 

Numerous researchers evidence the correlation between a good body image and the practice of physical activity and sport [11,12,13]. Despite this, there is still no scientific consensus that clarifies the influences of activity according to its typology and structuring. On the one hand, Ref. [31] states that “in sports with an aesthetic predominance, a negative load on body self-esteem usually prevails” (p. 71), degenerating and devaluing the body image of the subject. On the other hand, Ref. [32] clarifies that the practice of organized sports, such as football, and independently of their groupings, is associated with the possession of a positive body image. 

Football is considered a mass sport, which refers to a phenomenon of great social and cultural relevance that imprints great morale as well as aesthetic character in its various manifestations Ref, [33]. Authors such as [34] or [35] found that this sport of great popularity and universality is one of the preferred and most practiced activities by young people in Europe. Although it has been shown that playing football has important effects on the health of young people [36], we must go further. Football has become a way of distraction and recreation that, as [37] defend, ‘*generates communication processes from which senses of identification and recognition mechanisms are created that allow the configuration or recreation of identities, as well as the relationships, counterpositions and strengthening of these*’ (p. 1).

For these reasons, the practice of soccer as an extracurricular activity can be a turning point in the construction of the body image of schoolchildren [38]. The aim of this research is to identify the different factors that influence the positive social, negative social, and self-perception dimensions of the social construction of body image in fifth and sixth grade of Primary Education. For this reason, we address factors such as sex, number of siblings, and BMI, as well as factors related to context such as the practice of physical activity during extracurricular activities and football practice.

## 2. Materials and Methods

### 2.1. Sample Description

We performed a descriptive exploratory study with 719 pupils of 5th grade (*n* = 397; 55.2%) and 6th grade (*n* = 322; 44.8%) from Primary Education; 373 of them were boys (51.9%) and 340 girls (47.3%), with 6 lost samples (0.8%). In total, 10.4% (*n* = 75) of the individuals were only children, while 50.1% (*n* = 360) had one brother/sister; 23.1% (*n* = 166) had two brothers/sisters and 14.2% (*n* = 110) had at least three brothers/sisters. The average weight of these children was 40.51 kg (D.T. = 8.72), the height was approximately 148 cm (D.T. = 9.74), and BMI was 18.48 (D.T. = 2.99) on average. From the total amount of participants, 62.4% (*n* = 449) had physical activity as an extracurricular activity and 28.1% (*n* = 202) had played football for between 6 and 12 months (M = 13.48; D.T. = 25.02). 

### 2.2. Tools for Gathering Information 

Data gathering was performed through a questionnaire designed ad hoc for this research called “Child Body-Perception Questionnaire. CBPQ” and was inspired by previous research such as [39,40,41,42,43,44]. This research tool was composed of eight items that evaluate different aspects of body perception on a Likert scale of 5 points (1—disagree; 5—agree). These items were preceded by a series of questions related to the sex, height, and weight of the participants, as well as their grade, number of brothers and sisters, practice of physical education as an extracurricular activity, and whether this practice was football. 

The factorial structural of this instrument was checked after rejecting that the matrix of correlations between items did not form an identity matrix (Test KMO = 0.689; Bartlett’s Sphericity χ^2^ _28_ = 500.841, *p* < 0.001). An analysis through Factorization of Main Exes was made to analyze the shared variance, considering the error and knowing that the sample’s distribution was far from normal (Test K-S < 0.05; Levene Test < 0.05) [40]. Orthogonal Rotation with Varimax was used and coefficients <0.30 were suppressed because the sample was superior to 300 individuals [41], obtaining as a result a factorial structure composed by three factors that explained 38,88% of the total variance. The structure was made up of the three dimensions shown in Table 1.

As it is observed in Table 1, factors 1 and 3 are denominated social positive dimension and self-concept, respectively, and have three items each. The factors of the first one refer to body perception based on the evaluation of oneself. The second one contains items related to the child’s vision of their own self-perception and the importance of taking care of it. In this case, both items present a negative feature in relation to the factor’s relevance. This fact demonstrates that the relationship between items 1, 3, and 6 is an opposite relation because the bigger the increase in the importance of having a nice body and taking care of one’s image, the smaller the satisfaction with their body. Factor 2 contains the social negative valuations because it refers to the importance given by pupils to actions that might develop a negative perception about them from others.

McDonald’s omega (Ω) coefficient was used to estimate the reliability of both the overall instrument and its individual dimensions, as it does not assume tau-equivalence among items and offers more accurate estimates than Cronbach’s alpha, particularly in multifactorial structures and when the number of items per factor is limited [45]. The overall internal consistency was acceptable (Ω = 0.60), while lower values were observed in the *social positive* (Ω = 0.51), *social negative* (Ω = 0.44), and *self-concept* (Ω = 0.41) dimensions. Although the total omega would have been marginally improved by removing Item 6 (Ω = 0.61), this was not pursued due to the already limited number of items in the scale.

### 2.3. Procedure for Data Collection and Analysis

The data were collected during the 2019/2020 academic year in pre-schools and primary schools in the region of Albacete (Castilla-La Mancha). The information was collected directly in the classrooms, thus achieving a high rate of pupil participation. Then, the questionnaires obtained were filtered. Although no questionnaires were eliminated, there were some blank answers in some of the items, as well as in the response to the identification data. 

The data obtained were analyzed with the IBM SPSS Statistic 27 statistical package. The data were explored descriptively through the calculation of mean values and frequencies (according to the type of variables) and Chi-square for the contrast between variables, as well as inferentially, through non-parametric tests, given the non-compliance with the assumption of the normality (Kolmogorov–Smirnov goodness-of-fit test, *p* < 0.001) and homogeneity of variances (Levene’s test, *p* < 0.001). The Mann–Whitney U test for independent values and Spearman’s Rho were used to identify correlations with variables. A 95% confidence level (*p* < 0.05) was established to determine the presence of statistically significant differences [46]. For its interpretation, the conventional measures proposed by Cohen [47] were used, where a small effect size was established at d = 0.2, medium at d = 0.5, and large at d = 0.8. 

## 3. Results

The results of the descriptive and inferential analysis on identifying personal–contextual factors that influence the social construction of the body in fifth- and sixth-grade pupils are presented below.

### 3.1. Inferential and Descriptive Analysis of the Association Between Sex, Sports Practice, and Social Construction of Fifth- and Sixth-Grade Pupils in Primary Education

Table 1 identifies the distribution of extracurricular physical activity practice, football, and enjoyment of this practice according to sex. The data showed that the practice of extracurricular activity is slightly higher among boys compared to girls; this difference was not statistically significant (χ21 = 1.790, *p* = 0.181). More noticeable was the difference between boys and girls in football practice. Significantly more boys claimed to play football (χ21 = 106.24, *p* < 0.001) and to like playing football (χ21 = 54.45, *p* < 0.001). The strength of association between sex and football practice was moderate and positive (Crammer’s V = 0.386, *p* < 0.001), and between sex and liking to play football, it was weak and positive (Crammer’s V = 0.277, *p* < 0.001).

The data showed how extracurricular physical activity practice is slightly superior in boys compared to girls; this difference is not statistically significant (χ21 = 1.790, *p* = 0.181). The difference between boys and girls related to football practice is more noticeable because a bigger number of boys affirmed playing football (χ21 = 106.24, *p* < 0.001) and preferred the practice of this sport (χ21 = 54.45, *p* < 0.001) as it is shown in Table 2. The strength of association between sex and football practice was moderate and positive (Crammer’s V = 0.386, *p* < 0.001), and between sex and liking to play football, it was weak and positive (Crammer’s V = 0.277, *p* < 0.001).

Once the associations between sex and extracurricular physical activity practice were explored, it was relevant to considerate the influence of sex on the perception of the child’s body as well as this perception in relation to extracurricular physical activity practice.

As shown in Table 3, sex was an influential variable in the consideration of body image in terms of negative social perception (*p* = 0.008, d = 0.20) and self-concept (*p* = 0.018, d = 0.20) with a small effect size. Although it is not related to the positive social dimension, it was the girls who presented a significantly worse and negative perception of their body image than the boys. This was in line with what had already been described in the data with the percentile values, which placed 50% of the girls’ scores in lower values than boys. This situation was also observed in a positive social dimension, although it was not significant. In relation to sports practice, no differences were observed in the positioning of the subjects’ responses regarding their negative social perception and self-concept, but there were differences in the positive social perception (*p* = 0.014), where 75% of the responses of the subjects who did practice physical activity accumulated 0.5 points above those who did not practice physical activity. The magnitude of the effect of this relationship was small (d = 0.18). Football practice influenced all three dimensions of body perception. Positive social perception was significantly higher in those pupils who practiced football (*p* = 0.015, d = 0.17); they also gave more relevance to negative social perception (*p* = 0.020, d = 0.21), and this physical activity had an impact on their self-concept (*p* = 0.004, d = 0.25). The effect size of the associations was small (d < 0.5). It was not relevant, from a child’s body perception, to like football (*p* > 0.05).

A deeper analysis of all the items that are contained in the questionnaire have helped to identify the features that differentiate each infant’s body perception in relation to sex (see Table 4) from physical activity practice (see Table 5) and, specifically, football practice (see Table 6). In all cases, the percentiles show equivalent results in sex, physical education practice, and football practice in most variables. With the aim of facilitating the comparison between groups, information is accompanied with central tendency and dispersion statistics. Through these statistics, it has been possible to identify the differences in the infant’s body perception related to the contrast variables.

Table 4 observes how pupils positively valued the items related to their own body perception, except for items 4 (I dress in the same way as my classmates (I follow fashion)) and 5 (I wear on my body (necklaces, earrings, bracelets, piercings…) that I see on the TV or on the Internet), where girls obtained higher average values; the dispersion of their answers was bigger too.

The results obtained for Item 5 were statistically significant (*p* = 0.014), with a small effect size (d = 0.16), but not the ones related to Item 4 (*p* = 0.123), because their interpretation must be based on our participant sample’s context. Furthermore, Item 1 ‘Is positive to have a nice body (be attractive, be pretty) to be popular (having more Friends, they will love you more…) at school’ was significantly more valued by boys than by girls (*p* = 0.009, d = 0.21). The same happened with Item 2 ‘If you are good at sports, you will be more appreciated at school’ (*p* < 0.001, d = 0.37) and Item 7 ‘being too fat is something that schoolmates don’t like’ (*p* = 0.007, d = 0.21).

In the case of the practice of extracurricular activity linked or not to physical activity (see Table 5), it was found that, in general, those children who do not practice it scored higher in most items of the questionnaire. These differences are statistically significant in the case of Item 1 (Is positive to have a nice body (be attractive, be pretty) to be popular (having more Friends, they will love you more…) at school) (*p* = 0.034, d = 0.15) and Item 5 (I wear on my body (necklaces, earrings, bracelets, piercings…) that I see on the TV or on the Internet) (*p* < 0.001, d = 0.13).

On the other hand, Item 6 (I am satisfied with my body (I do not look too fat or too skinny…) was significantly better valued by those who were engaged in extracurricular physical activity (*p* = 0.003, d = 0.22) as it is linked to Item 7 (Being too fat is something that schoolmates don’t like); however, this difference was not significant (*p* = 0.558). Likewise, it was observed that the ratings made on Item 8 (Showing weakness (for example, crying…) is something that my schoolmates do not like) were equivalent.

Football practice, as can be seen in Table 6, influenced the valuations about body perception positively, except for body satisfaction. Those who indicated that they played football valued significantly more positively having a nice body to be popular at school (Item 1; *p* < 0.001, d = 0.23), being good at sports to be more appreciated (Item 2; *p* = 0.004, d = 0.21), the importance of taking care of their image (Item 3, *p* = 0.025; d = 0.20), and the perception that being fat is not liked by the rest of the classmates (Item 7; *p* = 0.007, d = 1.22). It is important to highlight the differences found in this last item because its magnitude of effect was high (d > 0.80).

### 3.2. Descriptive and Inferential Analysis of Body Mass Index, Number of Siblings, and Social Body Perception of Fifth- and Sixth-Grade Pupils in Primary Education

The study of the association between Body Mass Index (BMI: M = 18.48, DT = 2.99), the number of siblings (M = 1.51, DT = 1.09), and the social construction of the infant’s body was carried out through Spearman’s correlation analysis for the three considered dimensions and for each item individually (see Table 7).

In relation to the dimensions, it was found that only self-concept was associated with BMI (r = 0.134, *p* = 0.004, d = 0.37), where the higher the BMI, the greater the concern for body care and image. Observing the data explained in Table 7, it is easier to detail more precisely the information of this dimension by finding that BMI correlated positively and weakly (r = 0.139, *p* < 0.001), with a low magnitude of effect (d = 0.37), with the importance given to having a nice body to be popular (Item 1) and negatively weakly (r = 0.101, *p* = 0.016), with a low magnitude effect (d = 0.32), with satisfaction with their bodies. The association between BMI and the positive (r = 0.016, *p* = 0.699) and negative (r = 0.038, *p* = 0.371) social dimensions was not significant. Nevertheless, the association found with Item 8 (Showing weakness (for example, crying…) is something that my schoolmates do not like) was weakly positive (r = 0.135, *p* < 0.001) and with a small magnitude of effect (d = 0.38).

Finally, it was found that there is a statistically significant, positive, and weak association (r = 0.193, *p* < 0.001), with an effect size approaching a moderate value (d = 0.44), between the number of siblings and positive social perception. Again, attending to the data presented in Table 7, these associations can be identified in all the items of this dimension. An example is Item 2 (If you are good at sports, you will be more appreciated at school), for which the relationship was very weak (r = 0.075, *p* = 0.046), as was its magnitude of effect (d = 0.27).

Item 4 (I dress the same way as my school mates (I follow fashion)) had a weak and positive relationship (r = 0.145, *p* < 0.001) and a small effect size (d = 0.38). And, above all, Item 5 (I wear on my body (necklaces, earrings, bracelets, piercings…) that I see on the TV or on the Internet) had a moderate positive association (r = 0.296, *p* < 0.001) and a moderate effect size (d = 0.54). A positive and weak association (r = 0.098, *p* = 0.009) was also found for Item 1 (Is positive to have a nice body (be attractive, be pretty) to be popular (having more Friends, they will love you more…) at school), with a small effect size (d = 0.31), and a negative weak association (r = −0.149, *p* < 0.001) was found for Item 6 (I am satisfied with my body (I do not look too fat or too skinny), which had a small effect size (d = 0.38).

## 4. Discussion

This study focuses on the process of the social construction of infant’s bodies on Primary Education. Through this research, the researchers aimed to understand and identify the different personal and contextual factors that affect the positive social dimension, negative social dimension, and body self-concept construction. For this reason, the researchers determined a sample composed of 719 students from fifth and sixth grade of Primary Education from different schools in Albacete. After selecting the sample, the researchers carried out data collection by using an ad hoc questionnaire named “Body Perception Questionnaire”. After a descriptive and inferential analysis, the main findings suggested the existence of three key elements that provide widely relevant information on the influence of personal–contextual factors on children’s body image, which has been complemented with a more detailed study of the influence of these factors on each item of the applied instrument.

The study of the factorial structure of the instrument allowed us to focus on the three key elements in the perception of body image in children in the fifth and sixth grades of Primary Education: the positive social component, which incorporates and reflects the importance that the child attaches to the evaluations that others make of him/herself; the negative social component, which alludes to the relevance that these children give to avoiding those actions that could lead to a negative perception of others towards them; and the self-concept component, focused on analyzing the child’s self-perception of his/her own body image and the importance of taking care of it. Having verified in previous studies the interactions between children’s body perception and several personal and contextual variables [7,9,20,25], this research incorporated measures that allowed the researchers to identify the influence of some of them on the pupils.

In relation to the practice of physical activity by pupils in fifth and sixth grade of Primary Education, statistical significance was obtained in relation to the positive social dimension. The fact that those students who practice physical activity obtained lower scores (which were significant in several cases) in the evaluations of their body perception may be related to the conception that the practice of this physical activity is focused on recreation, leisure, and the enjoyment it produces, and not so much on the social or aesthetic aspect that is generated around the improvement of body image through sporting activity. In addition, according to [48], this generates a devalued image of the body and an increase in dissatisfaction and concern for the body shape, especially in the female sex. Furthermore, like in [13] or [14], this idea is reinforced by the identification of significantly higher values in Item 6, related to satisfaction with one’s own body, in those boys and girls who practiced physical activity.

Based on the presence of the greater practice of extracurricular physical activity, especially football, in boys compared to girls, gender was identified as an influential variable in negative social perception and in children’s self-concept. More specifically, it was found that, for boys, the image that others have of them in relation to their physical condition (being more appreciated for being good at sports and for having a good body; recognizing being fat as something socially negative) is more important than for girls, while, for girls, the influence seems to transcend beyond the peer group and is conditioned by the media (carrying on the body things they see in programs, movies, series…).

Football, referred to as a very popular sport, turned out to be a variable of interest when statistically significant differences were found in the three dimensions referring to the infant’s body. The practice of this sport was associated with a higher evaluation of the positive and negative social component and of the self-concept. This seems to show that practicing this sport not only represents a way of keeping active and in shape, but may also be conditioning the way in which the child sees him/herself, and the perception he/she has of how others see him/her.

BMI was related to the perception of the child’s body in that, as BMI increased, concern for body image, for not appearing weak in front of classmates increased and satisfaction with the body decreased. The bigger the number of siblings, the bigger the consideration of social and cultural aspects that can improve body image (dressing by following fashion and using accessories that are advertised on TV) and the perception that others have on people (to have a nice body to be appreciated or to be good at sports). For these reasons, the fact that having more siblings decreases the infant’s satisfaction with his/her own body was significant.

### Limitations and Further Research

The results of this research show a clear relationship between sports practice and the perception of body image, in this case, football, when it is practiced as an extracurricular activity. Studies highlight how positive its impact can be for football players not only on their body image perception, but on what is around it: daily habits, nutrition, and even how they perceive their environment in terms of health and well-being even at young ages [49]. However, there are some limitations that might serve as future lines of research such as the fact of having used a sample centered in a region of Spain, instead of doing so on a sample at the national or even transnational level. In view of the results achieved, having differentiated three key elements in the perception of child body image (positive social component, negative social component, and self-concept), the need to continue to advance in the study of contextual factors that have an impact on the child’s self-image in different learning environments becomes evident.

Secondly, the fact that this study focused on one sport, football, cannot be ignored. Therefore, it is necessary to check whether or not the perception that the pre-teen has of their body image could be different before practicing any other type of sport such as basketball, tennis, swimming, etc., to see whether the results are similar or not. As recent studies have shown, football remains an important field of research due to how its practice influences both boys’ and girls’ body image [50]. Finally, there is a need to increase the number of individual variables (emotions, satisfaction with life, self-evaluation…) as well as contextual variables (family, school, and socio-community) in a way that allows for a broader and more complete view of how children build body image.

Furthermore, it is important to highlight the biggest limitation of this research, which was found in the analysis. While the internal consistency of the overall scale was acceptable (Ω = 0.60), lower reliability coefficients were observed in the subscales, which can be partly attributed to the small number of items per factor (2–3 items). Previous research has noted that omega values below 0.70 may still be considered acceptable in early-stage or exploratory studies, particularly when using brief scales [50]. Furthermore, the conceptual relevance of all items to the construct under study supported their retention, even when item removal slightly improved global reliability.

For future research, it is important to consider that, in the social construction of infants’ bodies in Primary Education, there are numerous variables which depend on family decisions, such as participation in physical activities (especially in football) and different factors such as BMI or having siblings. It is also important to try to know and determine possible indirect influences that can affect families in their perception of the body image of their sons and daughters. Due to the new character of this field of study, these findings could stimulate the beginning of new lines of research to help researchers to better understand the role of influencers and their relationship with body image. Furthermore, it is important to highlight how important it is to develop a positive construction of body image at young ages, as it clearly influences other developmental processes in the teenage years and the social relationships of teenagers [17].

## 5. Conclusions

The present study significantly contributed to increasing our knowledge on the social construction of body image in childhood. The main findings of this research help to reinforce the relationships between the lack of physical activity practice, increased BMI, and increased number of siblings with the increased self-perception of their bodies. Likewise, while the practice of extracurricular activities indicated a strong contrast between negative social perception and infants’ self-perception according to sex, the practice of football was one of the most interesting variables regarding the three dimensions referring to children’s bodies.

Therefore, football as an extracurricular activity is understood as a factor that conditions the way in which boys and girls see themselves in the fifth and sixth grades of Primary Education. This perception is also related to how others see them. These findings could stimulate the beginning of new lines of research to help better understand the role of football and family decisions and their possible influences on children’s bodies.

## Figures and Tables

**Table 1:** Factorial structure of the questionnaire on perception of the infant’s body.

Dimension	Item	Factor		
		1	2	3
Social positive	4. I dress the same way as my schoolmates (I follow fashion).	0.652		
	2. If you are good at sports, you will be more appreciated at school.	0.416		
	5. I wear on my body (necklaces, earrings, bracelets, piercings…) that I see on the TV or on the Internet.	0.344		
Social negative	8. Showing weakness (for example, crying…) is something that my schoolmates do not like.		0.624	
	7. Being too fat is something that schoolmates don’t like.		0.426	
Self-concept	1. Is positive to have a nice body (be attractive, be pretty) to be popular (having more Friends, they will love you more…) at school.			0.456
	3. I try to take care of my image (choosing nice clothes as an example…).			0.368
	6. I am satisfied with my body (I do not look too fat or too skinny…).			−0.305

**Table 2:** Analysis of the associations between sports practice and sex.

	SEX					
	Boys		Girls		Total	
	**N**	**%**	**N**	**%**	**N**	**%**
Practice of extracurricular activities linked to: Physical activity	243	54.2	205	45.8	448	100
Football practice	167	83.1	34	16.9	201	100
Taste for football	312	60.9	200	39.1	512	100

**Table 3:** Analysis of the influence of sex and extracurricular physical activity practice (football) on body perception.

		Social Positive					Social Negative					Self-Concept				
		P_25_	P_50_	P_75_	U	Z	P_25_	P_50_	P_75_	U	Z	P_25_	P_50_	P_75_	U	Z
Sex	H	1.75	2.25	3	59,950.5	−1.21	3	3	3	5609.0	−2.66 *	1.5	2.5	3	56,844.5	−2.36 *
	M	1.5	2.25	2.93			3	3	3.50			1	2	3		
Physical activity practice	Si	1.75	2.25	2.75	52,631.0	−2.46 *	3	3	3.5	5894.0	−0.071	1	2.5	3	57,407.5	−0.663
	No	1.75	2.25	3			3	3	3.5			1	2.5	3		
Taste for football	Si	1.75	2.5	3	177,039.0	−2.43 *	3	3	4	46,142.0	−2.32 *	1.5	2.5	3.13	176,022.0	−2.87 *
	No	1.75	2.25	2.75			3	3	3.5			1	2	3		
	Si	1.75	2.25	2.88	49,843.0	−0.490	3	3	4	48,017.0	−1.27	1	2.5	3	49,710.0	−0.550
	No	1.75	2.25	3			3	3	4			1.5	2	3		

* *p* < 0.05.

**Table 4:** Item analysis related to sex influence on body perception.

	Men (*n* = 371)					Women (*n* = 337)						
	M	DT	P_25_	P_50_	P_75_	M	DT	P_25_	P_50_	P_75_	U	Z
Item 1	2.41	1.42	1	2	3	2.13	1.30	1	2	3	56,438.5	−2.61 *
Item 2	2.41	1.36	1	2	3	1.91	1.34	1	1	3	49,474.5	−5.34 **
Item 3	3.26	1.39	2	3	4	3.20	1.39	2	3	4	61,368.0	−0.631
Item 4	2.06	1.27	1	2	3	2.22	1.31	1	2	3	59,248.5	−1.54
Item 5	1.68	1.21	1	1	2	1.88	1.45	1	1	2	57,352.0	−2.46 *
Item 6	4.14	1.21	3	5	5	4.09	1.26	3	5	5	61,945.0	−0.451
Item 7	2.42	1.44	1	2	3	2.13	1.34	1	2	3	56,217.5	−2.71 *
Item 8	2.54	1.47	1	2	4	2.41	1.41	1	2	4	59,601.0	−1.31

* *p* < 0.05; ** *p* < 0.001.

**Table 5:** Item-by-item analysis of the influence of extracurricular physical activity on body perception.

	Extracurricular Activity Linked to Physical Activity (*n* = 447)					Extracurricular Activity Not Linked with Physical Activity (*n* = 268)						
	**M**	**DT**	**P_25_**	**P_50_**	**P_75_**	**M**	**DT**	**P_25_**	**P_50_**	**P_75_**	**U**	**Z**
Item 1	2.19	1.39	1	2	3	2.40	1.37	1	2	3	54,638.0	−2.12 *
Item 2	2.08	1.24	1	2	3	2.31	1.56	1	2	3	56,376.5	−1.45
Item 3	3.20	1.39	2	3	4	3.26	1.39	2	3	4	58,259.0	−0.678
Item 4	2.09	1.25	2	3	4	2.18	1.34	2	3	4	58,838.5	−0.473
Item 5	1.69	1.37	1	2	4	1.91	1.26	1	3	4	52,505.5	−3.25 **
Item 6	4.22	1.18	5	5	5	3.94	1.31	4	5	5	52,666.0	−2.97 *
Item 7	2.31	1.42	1	2	3	2.22	1.37	1	2	3	5852.5	−0.585
Item 8	2.50	1.45	1	2	4	2.46	1.43	1	2	3	58,968.0	−718

* *p* < 0.05; ** *p* < 0.001.

**Table 6:** Analysis by items of the influence of football practice on body perception.

	Plays Football as an Extracurricular Activity (*n* = 202)					Does Not Play Football as an Extracurricular Activity (*n* = 516)						
	M	DT	P_25_	P_50_	P_75_	M	DT	P_25_	P_50_	P_75_	U	Z
Item 1	2.5	1.38	1	2	4	2.18	1.36	1	2	3	44,527.5	−3.19 **
Item 2	2.37	1.37	1	2	3	2.08	1.36	1	1	3	45,341.0	−2.88 *
Item 3	3.41	1.32	3	4	5	3.14	1.41	2	3	4	46,541.5	−2.25 *
Item 4	3.14	1.41	1	2	3	2.18	1.28	1	2	3	49,734.5	−1.01
Item 5	2.10	1.29	1	1	2	1.69	1.11	1	1	2	51,306.0	−0.261
Item 6	1.81	1.41	3	5	5	4.14	1.15	3	5	5	51,432.5	−0.260
Item 7	4.11	1.27	1	2	3	2.48	1.40	1	2	3	45,744.5	−2.70 *
Item 8	2.2	1.39	1	3	4	2.67	1.42	1	2	3	46,024.0	−2.495 *

* *p* < 0.05; ** *p* < 0.001.

**Table 7:** Analysis of the correlations between BMI, number of siblings, and social construction of infant’s body on fifth- and sixth-grade pupils in Primary Education.

		BMI	Siblings	Item 1	Item 2	Item 3	Item 4	Item 5	Item 6	Item 7	Item 8
BMI (*n* = 569)	Rho	1.000		0.139 **	0.052	0.001	−0.031	0.024	−0.101 *	0.070	0.135 **
	*p*	.		0.001	0.211	0.977	0.465	0.574	0.016	0.093	0.001
Siblings (*n* = 703)	Rho		1.000	0.098 **	0.075 *	0.019	0.145 **	0.296 **	−0.149 **	0.012	−0.040
	*p*		.	0.009	0.046	0.608	0.000	0.000	0.000	0.759	0.293

*Note*: * *p* < 0.05; ** *p* < 0.001.

## Data Availability

The data that supports the findings of this study are available from the corresponding author, M.M., upon reasonable request. The data are not publicly available due to privacy and ethical restrictions as they involve children.
